# Supplementary material for: Protocol for a scoping review of the evidence concerning the unique needs and experiences of Orthodox Jewish women and their partners using maternity services
Source: PLoS One. 2025 Aug 6;20(8):e0323838. doi: 10.1371/journal.pone.0323838 (PMC12327656; doi:10.1371/journal.pone.0323838)
Supplement: Attachment — Submitted filename: PLOS One 6.2025.docx [file pone.0323838.s001.docx]

**Protocol for a scoping review of the evidence concerning the unique needs and experiences of Orthodox Jewish women and their partners using maternity services in the UK**

**Reviewer comments:**

- This protocol is on an interesting topic that will no doubt be important to understand and improve the care of the Orthodox Jewish community in the UK. However, upon reviewing the types of articles that PLOS One publishes, it doesn’t seem that a protocol for a scoping review fits what PLOS One is looking for. I am still including my feedback for this paper if the editor decides that it will be accepted.

Introduction:

- Page 3, line 77: you introduce the word “shabbat.” This is the Hebrew term that readers may not be familiar with. You may want to consider introducing it as “the Sabbath” and then in parenthesis, saying (Shabbat/Shabbos).
- You established a good background and gap in the literature and really give the reader a good look into why this work is important. I am wondering if there is any literature you could cite to bolster this regarding whether Orthodox women experience more or high rates of maternity complications compared to the general population in the UK?

Methods:

- The acronym PPI work has been used several times, but it is unclear what it stands for. Please introduce the acronym upon first use, and then use the acronym.
- Grey literature also includes PhD dissertations and thesis, are you including those? If so, be mindful of how long they are for the full-text review and data extraction process.
- Are you planning on using any software, such as Covidence, to house and track your articles and data extraction? If so, please state that in the methods.
- I am worried that given your timeframe of 6 months, you may be biting off more than you can chew by expanding your search to outside of the UK. As you describe in the methods, your research questions are specific to the UK, as your paper title also states. The purpose of a scoping review is to map the data to then guide future work. I understand that your initial search did not turn up many results, but THAT is your gap! Mapping the few studies that have been done will then allow you to discuss the fact that there are many gaps in the literature, and they are x, y, and z based on the findings of your scoping review.
- In scoping reviews, data extraction should first be done separately and independently by 2 people and then compared for consensus. With the shared Excel file, how can you guarantee independent extraction is being done?

Discussion:

- Again, you discuss this review is to identify needs of Orthodox Jewish women in the UK, but if you include literature from Israel, you may get a very different view of needs. Israel has a large Jewish and Orthodox Jewish population, and therefore, many healthcare professionals are likely sensitive to the needs of these patients. In the UK, as you stated, the Orthodox community doesn’t interact much with the external world, so their experience in healthcare situations will likely be different.

In general, I am excited to read the results of your review and learn about your findings.
